# Action and problems related to the COVID-19 outbreak in India

**DOI:** 10.1017/ice.2020.186

**Published:** 2020-05-04

**Authors:** Pooja Sharma, Karan Veer

**Affiliations:** 1 Department of Instrumentation & Control Engineering, Dr B. R. Ambedkar National Institute of Technology, Jalandhar, Punjab, India

*To the Editor—* Named SARS-CoV-2 by the International Committee on Taxonomy of Viruses, this novel coronavirus attacks the lower respiratory tract of patients infected with cryptogenic organizing pneumonia.^1^ The infectious disease it causes was named COVID-19 by the World Health Organization. Coronaviruses comprise a large family of viruses that cause illness ranging from the common cold to more severe diseases like pneumonia, Middle East respiratory syndrome (MERS), and severe acute respiratory syndrome (SARS). Most people are vulnerable to SARS-CoV-2, and this novel coronavirus can affect people with low or normal immunity.^2^ People with low immunity, such as the elderly, pregnant women, and patients with chronic diseases, are prone to severe acute symptoms after contracting COVID-19. SARS-CoV-2 is mainly transmitted via droplets, touching (including self-infection caused by contaminated hands), and short-distance transmission of respiratory aerosols of different sizes. Currently, SARS-CoV-2 is mainly spread via droplets. At first, this virus was transferred from bats to humans; it falls into a specific category of bat viruses. Different coronaviruses persist on surfaces for various lengths of time.

**Fig. 1.**
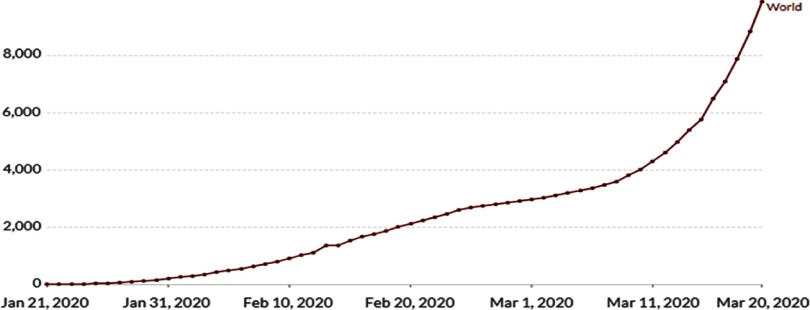
Death rate increment due to COVID-2019 throughout the world.^5^

As 2019 ended, news arrived of an epidemic of pneumonia, with a few cases in a seafood wholesale market in Wuhan, China. Initially, a few cases were detected around December 8, and a cluster was revealed on approximately December 31, 2019, when the WHO office in China was given the information. The market was shut down on January 1, 2020, and the Chinese authority announced the viral threat. All active and suspected cases were tested. At that time, ~300 cases were positive and 4 people had died. Initially, few reports verified human-to-human transmission, and reports of super-spreading patients included 15 healthcare workers and viral spread to different Chinese cities. Various other countries also confirmed human-to-human transmission. After China, SARS-CoV-2 spread to Europe, across Asia, and throughout the rest of the world. On January 31, 2020, first case of COVID-19 was confirmed in Kerala, India, where a student tested positive as she returned from Wuhan, China.^3,4^ Presently, SARS-CoV-2 is still spreading throughout the world and has affected nearly 132,758 persons globally in 167 countries. Throughout the world, the death rate is extremely high (Fig. 1, as of March 20, 2020).

COVID-19 has been declared a national disaster by the Indian government.^6,7^ The scientists at the Indian Council of Medical Research (ICMR) are continually obtaining global information related to the pandemic. They suggest the use of retroviral drugs. The ICMR is providing free and reliable testing and diagnosis to all individuals with symptoms of COVID-19. The government is trying to expand laboratory testing using Ministry of Health and Family Welfare (MOHFW) and non-ICMR laboratories in many facilities and organizations, such as the Council of Scientific & Industrial Research (CSIR), the Department of Biotechnology (DBT), Defence Research, and the Development Organization (DRDO), and government medical colleges. Thus far, 15 laboratories in India are testing for SARS-CoV-2, and 19 will soon be added.^8^ The agencies in India conducting COVID-19 testing include the National Institute of Virology (NIV) in Pune, the Indian Council of Medical Research (ICMR) in Hyderabad, and the National Center for Disease Control (NCDC) in Delhi. All of these agencies work under the NIV. A fund named the COVID Fund for South Asian Association for Regional Cooperation (SAARC) Countries has been started by SAARC countries to fight COVID-19. In addition, the Indian government has appealed to its citizens to follow social distancing procedures, which is the most effective way to stop the community transmission of SARS-CoV-2.

The current COVID-19 situation has affected the whole world and has had a dramatic impact on India. In India, the death rate is comparatively good, but the recovery rate of infected persons is not, which is leading to a difficult situation in India. Infections are increasing day by day in India, even though community transmission began only recently. The Indian government has taken a few necessary steps to control the situation, such as making masks and sanitizer available and providing free testing and diagnosis. Public awareness and programs of “do’s and don’ts” for COVID-19 are run at public places. Environmental conditions may also support controlling SARS-CoV-2; across Asia spring temperatures are increasing, which may decrease viral spread somewhat. Early prediction methods and a specific vaccine are not yet available, although government has been able to control the pandemic thus far. The World Health Organization (WHO) helps developing countries by providing funding, medical kits for testing, and proper guidance for treatment and safety. In India, the death rate and the recovery rate indicate that the pandemic is being controlled, largely because of the preparation done by government before COVID-19 reached more advanced stages. The numbers of laboratories, test kits, and medical facilities have been enhanced appropriately. The Indian government is collaborating with SAARC countries to fight this pandemic. Because the Indian government has taken the appropriate actions outlined here, the COVID-19 pandemic, although tragic, will have the best possible outcome in India.
